# Draft Genome of Escherichia coli O146 Isolate from Maulana Azad Medical College, New Delhi, India

**DOI:** 10.1128/genomeA.01515-14

**Published:** 2015-02-05

**Authors:** K. Rajeshwari, Beena Uppal, Rakesh Singh, Abhishek K. Malakar, Surendra K. Chikara

**Affiliations:** a Maulana Azad Medical College, New Delhi, India; b National Research Centre on DNA Fingerprinting, NBPGR, New Delhi, India; c Xcelris Genomics Ltd., Ahmedabad, Gujarat, India

## Abstract

Here, we report the draft genome sequence of enteropathogenic Escherichia coli (EPEC) O146 strain isolated from a 1-year-old child with acute diarrhea in Delhi who recovered completely. The multidrug transporter (*mdtABCD*) gene, responsible for drug resistance, is present. The strain also contains the *astA* gene, an additional virulence determinant.

## GENOME ANNOUNCEMENT

Here, we report the draft genome sequence of Escherichia coli polyvalent 2 O146 isolated from a child with acute diarrhea cultured at the enterobacteriaceae laboratory of the department of Microbiology at Maulana Azad Medical College. The infant was admitted to the diarrhea ward of Lok Nayak Hospital affiliated to Maulana Azad Medical College, a tertiary care teaching hospital in northern India. Its genome size was determined to be 5 Mb, and its genomic features along with a potential gene involved in virulence and multidrug resistance were analyzed.

Enteropathogenic E. coli (EPEC) infects children in developing countries causing diarrhea (1–3). EPEC adheres to the epithelial cells of the intestine which results in either watery or bloody diarrhea which is a consequence of acute tissue destruction. Secretion of effector molecules allows pathogenic bacteria to interact with their host and cause disease. The strain enteropathogenic E. coli polyvalent 2 O146 was isolated from a 1-year-old male infant admitted with acute watery diarrhea and vomiting for 24 h. The child recovered and was discharged.

The whole-genome sequencing of enteropathogenic E. coli was performed using Illumina NextSeq 500 at Xcelris Labs, Ltd., Ahmedabad. A total of 947.4 Mb of data was generated using a paired-end library with an average read length of 2 × 150 bp. The reads were quality filtered using Trimmomatic v0.30 with the following parameters: SLIDINGWINDOW-20: 20, LEADING: 20, TRAILING: 20, MINLENGTH: 50 bp, and adapter trimming. *De novo* assembly using Velvet v1.2.10 with a *k*-mer length of 131 and exp_cov auto and cov_cutoff auto parameters generated 188 scaffolds with a total assembly size of 4,954,160 bp (~5 Mb) and a scaffold *N*_50_ of 92,999 bp. The genome was annotated using the Rapid Annotation using Subsystem Technology (RAST) server (4). A total of 4,786 potential coding sequences (CDS) were identified, which included 794 hypothetical proteins and 113 RNA genes. Out of 113 RNA genes, 66 tRNA genes and 47 rRNA genes were identified.

Agglutination using antisera confirmed the O146 serotype and was identified as EPEC by the presence of the locus of enterocyte effacement (LEE) and the *bfp* gene. Attaching and effacing E. coli are characterized by the presence of the LEE pathogenicity island (5) and express the EPEC adherence factor (EAF) plasmid-carried bundle-forming pilus gene (*bfp*) (6). EPEC has been associated with attaching and effacing (A/E) and intestinal lesions which include secreted effector molecules, namely, bundle-forming pili (BFP) and *EspC* and *eae* genes (7, 8). There are also genes involved in tight association of pathogens with the host cell, virulence, hemolysin, sec-dependent release of pullulanase, and multidrug resistance (9). There are different secretion mechanisms (type I, II, and III) responsible for transfer of secretory products across the cytosolic (inner) and outer membranes in addition to the intervening periplasmic space. The multidrug transporter (*mdtABCD*) gene, responsible for drug resistance, is present (10). The *astA* gene, an additional virulence determinant, is also present.

This EPEC strain expressed virulence factors encoded by LEE and BFP regions and the *astA* gene. This is the first report of whole-genome sequence of E. coli O146 expressing a virulent and multidrug resistance gene.

### Nucleotide sequence accession number.

This whole-genome shotgun project has been deposited at DDBJ/EMBL/GenBank under the accession no. JWHN00000000.
